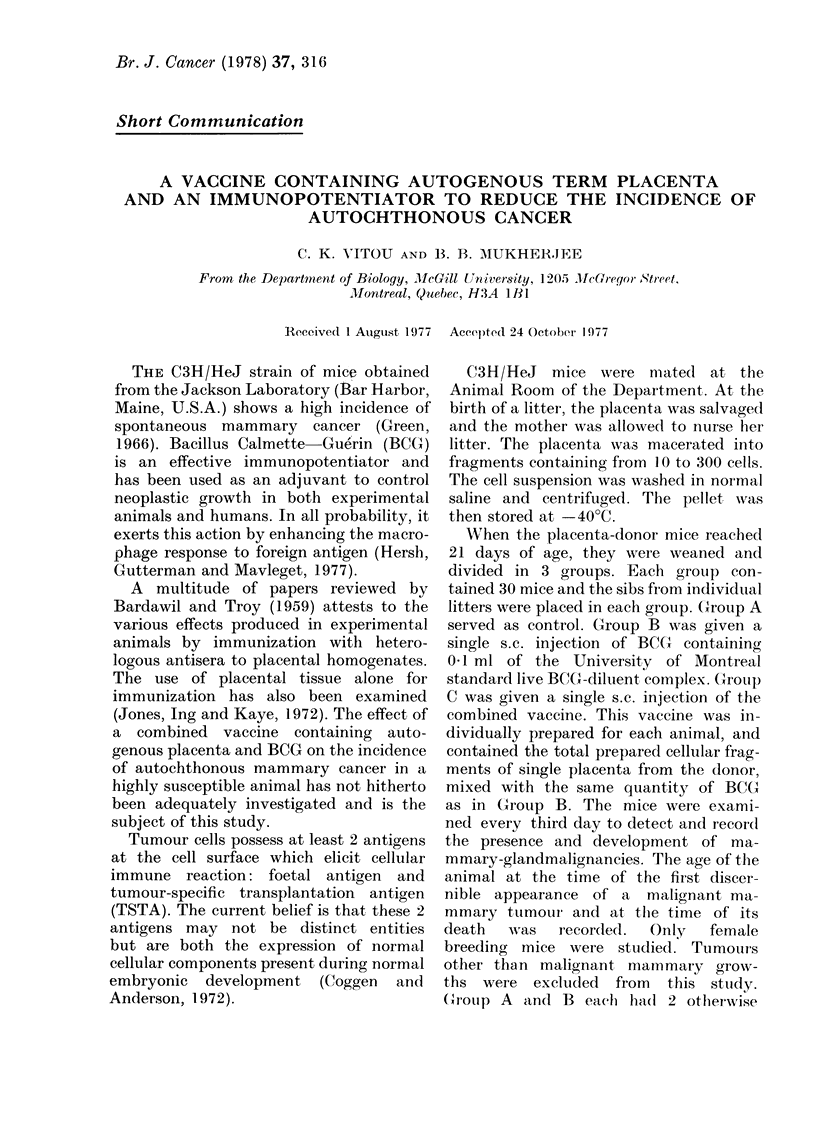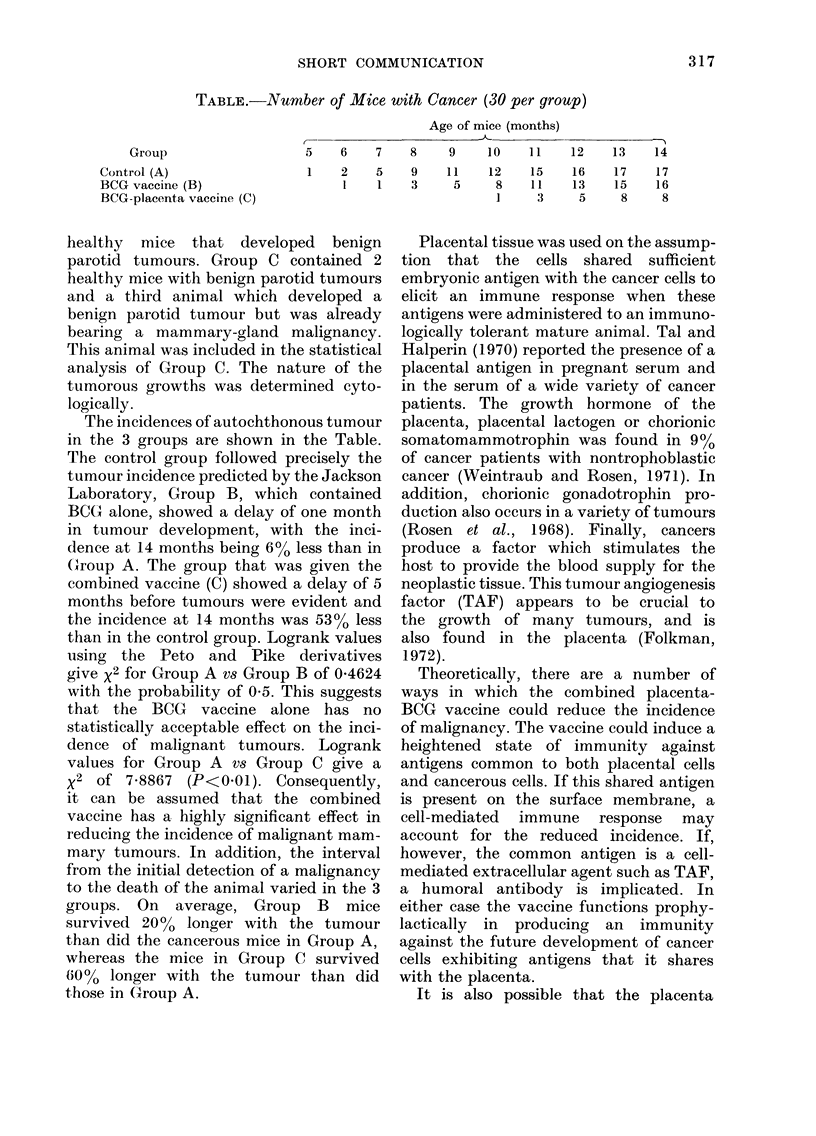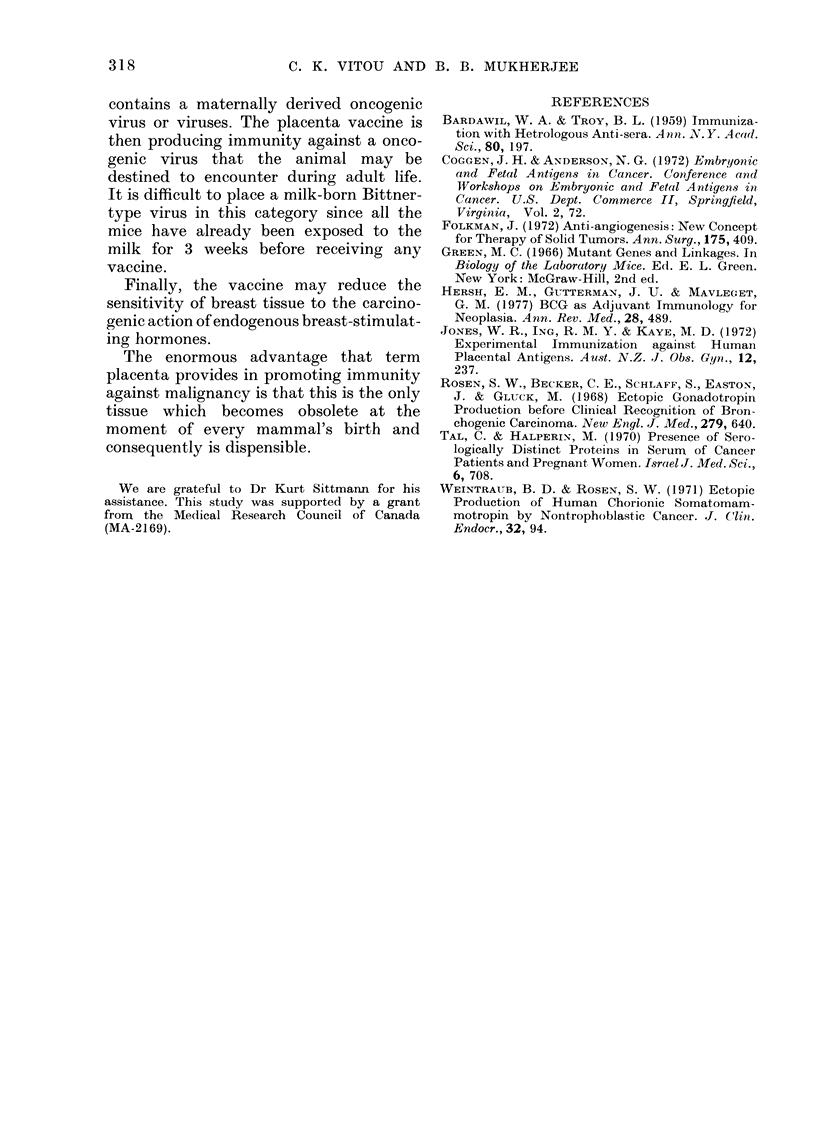# A vaccine containing autogenous term placenta and an immunopoteniator to reduce the incidence of autochthonous cancer.

**DOI:** 10.1038/bjc.1978.41

**Published:** 1978-02

**Authors:** C. K. Vitou, B. B. Mukherjee


*Br. J. Cancer* (1978) **37**, 316

***Short Communication***

### A VACCINE CONTAINING AUTOGENOUS TERM PLACENTA AND AN IMMUNOPOTENTIATOR TO REDUCE THE INCIDENCE OF AUTOCHTHONOUS CANCER

C. K. VITOU AND B. B. MUKHERJEE

*From the Department of Biology, McGill University, 1205 McGregor Street, Montreal, Quebec, H3A 1B1*

Received 1 August 1977 Accepted 24 October 1977

THE C3H/HeJ strain of mice obtained from the Jackson Laboratory (Bar Harbor, Maine, U.S.A.) shows a high incidence of spontaneous mammary cancer (Green, 1966). Bacillus Calmette—Guérin (BCG) is an effective immunopotentiator and has been used as an adjuvant to control neoplastic growth in both experimental animals and humans. In all probability, it exerts this action by enhancing the macrophage response to foreign antigen (Hersh, Gutterman and Mavleget, 1977).

A multitude of papers reviewed by Bardawil and Troy (1959) attests to the various effects produced in experimental animals by immunization with heterologous antisera to placental homogenates. The use of placental tissue alone for immunization has also been examined (Jones, Ing and Kaye, 1972). The effect of a combined vaccine containing autogenous placenta and BCG on the incidence of autochthonous mammary cancer in a highly susceptible animal has not hitherto been adequately investigated and is the subject of this study.

Tumour cells possess at least 2 antigens at the cell surface which elicit cellular immune reaction: foetal antigen and tumour-specific transplantation antigen (TSTA). The current belief is that these 2 antigens may not be distinct entities but are both the expression of normal cellular components present during normal embryonic development (Coggen and Anderson, 1972).

C3H/HeJ mice were mated at the Animal Room of the Department. At the birth of a litter, the placenta was salvaged and the mother was allowed to nurse her litter. The placenta was macerated into fragments containing from 10 to 300 cells. The cell suspension was washed in normal saline and centrifuged. The pellet was then stored at −40°C.

When the placenta-donor mice reached 21 days of age, they were weaned and divided in 3 groups. Each group contained 30 mice and the sibs from individual litters were placed in each group. Group A served as control. Group B was given a single s.c. injection of BCG containing 0·1 ml of the University of Montreal standard live BCG-diluent complex. Group C was given a single s.c. injection of the combined vaccine. This vaccine was individually prepared for each animal, and contained the total prepared cellular fragments of single placenta from the donor, mixed with the same quantity of BCG as in Group B. The mice were examined every third day to detect and record the presence and development of mammary-glandmalignancies. The age of the animal at the time of the first discernible appearance of a malignant mammary tumour and at the time of its death was recorded. Only female breeding mice were studied. Tumours other than malignant mammary growths were excluded from this study. Group A and B each had 2 otherwise

TABLE.—*Number of Mice with Cancer (30 per group)*

| Group | Age of mice (months) 5 | 6 | 7 | 8 | 9 | 10 | 11 | 12 | 13 | 14 |
|---|---|---|---|---|---|---|---|---|---|---|
| Control (A) | 1 | 2 | 5 | 9 | 11 | 12 | 15 | 16 | 17 | 17 |
| BCG vaccine (B) | | 1 | 1 | 3 | 5 | 8 | 11 | 13 | 15 | 16 |
| BCG-placenta vaccine (C) | | | | | | 1 | 3 | 5 | 8 | 8 |

healthy mice that developed benign parotid tumours. Group C contained 2 healthy mice with benign parotid tumours and a third animal which developed a benign parotid tumour but was already bearing a mammary-gland malignancy. This animal was included in the statistical analysis of Group C. The nature of the tumorous growths was determined cytologically.

The incidences of autochthonous tumour in the 3 groups are shown in the Table. The control group followed precisely the tumour incidence predicted by the Jackson Laboratory, Group B, which contained BCG alone, showed a delay of one month in tumour development, with the incidence at 14 months being 6% less than in Group A. The group that was given the combined vaccine (C) showed a delay of 5 months before tumours were evident and the incidence at 14 months was 53% less than in the control group. Logrank values using the Peto and Pike derivatives give $\chi^2$ for Group A *vs* Group B of 0·4624 with the probability of 0·5. This suggests that the BCG vaccine alone has no statistically acceptable effect on the incidence of malignant tumours. Logrank values for Group A *vs* Group C give a $\chi^2$ of 7·8867 ($P<0{\cdot}01$). Consequently, it can be assumed that the combined vaccine has a highly significant effect in reducing the incidence of malignant mammary tumours. In addition, the interval from the initial detection of a malignancy to the death of the animal varied in the 3 groups. On average, Group B mice survived 20% longer with the tumour than did the cancerous mice in Group A, whereas the mice in Group C survived 60% longer with the tumour than did those in Group A.

Placental tissue was used on the assumption that the cells shared sufficient embryonic antigen with the cancer cells to elicit an immune response when these antigens were administered to an immunologically tolerant mature animal. Tal and Halperin (1970) reported the presence of a placental antigen in pregnant serum and in the serum of a wide variety of cancer patients. The growth hormone of the placenta, placental lactogen or chorionic somatomammotrophin was found in 9% of cancer patients with nontrophoblastic cancer (Weintraub and Rosen, 1971). In addition, chorionic gonadotrophin production also occurs in a variety of tumours (Rosen *et al.*, 1968). Finally, cancers produce a factor which stimulates the host to provide the blood supply for the neoplastic tissue. This tumour angiogenesis factor (TAF) appears to be crucial to the growth of many tumours, and is also found in the placenta (Folkman, 1972).

Theoretically, there are a number of ways in which the combined placenta-BCG vaccine could reduce the incidence of malignancy. The vaccine could induce a heightened state of immunity against antigens common to both placental cells and cancerous cells. If this shared antigen is present on the surface membrane, a cell-mediated immune response may account for the reduced incidence. If, however, the common antigen is a cell-mediated extracellular agent such as TAF, a humoral antibody is implicated. In either case the vaccine functions prophylactically in producing an immunity against the future development of cancer cells exhibiting antigens that it shares with the placenta.

It is also possible that the placenta contains a maternally derived oncogenic virus or viruses. The placenta vaccine is then producing immunity against a oncogenic virus that the animal may be destined to encounter during adult life. It is difficult to place a milk-born Bittner-type virus in this category since all the mice have already been exposed to the milk for 3 weeks before receiving any vaccine.

Finally, the vaccine may reduce the sensitivity of breast tissue to the carcinogenic action of endogenous breast-stimulating hormones.

The enormous advantage that term placenta provides in promoting immunity against malignancy is that this is the only tissue which becomes obsolete at the moment of every mammal's birth and consequently is dispensible.

We are grateful to Dr Kurt Sittmann for his assistance. This study was supported by a grant from the Medical Research Council of Canada (MA-2169).

REFERENCES

BARDAWIL, W. A. & TROY, B. L. (1959) Immunization with Hetrologous Anti-sera. *Ann. N.Y. Acad. Sci.*, **80**, 197.

COGGEN, J. H. & ANDERSON, N. G. (1972) *Embryonic and Fetal Antigens in Cancer. Conference and Workshops on Embryonic and Fetal Antigens in Cancer. U.S. Dept. Commerce II, Springfield, Virginia*, Vol. 2, 72.

FOLKMAN, J. (1972) Anti-angiogenesis: New Concept for Therapy of Solid Tumors. *Ann. Surg.*, **175**, 409.

GREEN, M. C. (1966) Mutant Genes and Linkages. In *Biology of the Laboratory Mice.* Ed. E. L. Green. New York: McGraw-Hill, 2nd ed.

HERSH, E. M., GUTTERMAN, J. U. & MAVLEGET, G. M. (1977) BCG as Adjuvant Immunology for Neoplasia. *Ann. Rev. Med.*, **28**, 489.

JONES, W. R., ING, R. M. Y. & KAYE, M. D. (1972) Experimental Immunization against Human Placental Antigens. *Aust. N.Z. J. Obs. Gyn.*, **12**, 237.

ROSEN, S. W., BECKER, C. E., SCHLAFF, S., EASTON, J. & GLUCK, M. (1968) Ectopic Gonadotropin Production before Clinical Recognition of Bronchogenic Carcinoma. *New Engl. J. Med.*, **279**, 640.

TAL, C. & HALPERIN, M. (1970) Presence of Serologically Distinct Proteins in Serum of Cancer Patients and Pregnant Women. *Israel J. Med. Sci.*, **6**, 708.

WEINTRAUB, B. D. & ROSEN, S. W. (1971) Ectopic Production of Human Chorionic Somatomammotropin by Nontrophoblastic Cancer. *J. Clin. Endocr.*, **32**, 94.